# Mitigating RF Front-End Nonlinearity of Sensor Nodes to Enhance Spectrum Sensing

**DOI:** 10.3390/s16121999

**Published:** 2016-11-25

**Authors:** Lin Hu, Hong Ma, Hua Zhang, Wen Zhao

**Affiliations:** School of Electronic Information and Communications, Huazhong University of Science & Technology, Wuhan 430074, China; hulin0408@163.com (L.H.); mahong@hust.edu.cn (H.M.); jingtian_xuejian@126.com (W.Z.)

**Keywords:** cognitive radio wireless sensor network (CR-WSN), spectrum sensing, nonlinear distortion, singular value decomposition (SVD), adaptive interference cancellation (AIC)

## Abstract

The cognitive radio wireless sensor network (CR-WSN) has gained worldwide attention in recent years for its potential applications. Reliable spectrum sensing is the premise for opportunistic access to sensor nodes. However, as a result of the radio frequency (RF) front-end nonlinearity of sensor nodes, distortion products can easily degrade the spectrum sensing performance by causing false alarms and degrading the detection probability. Given the limitations of the widely-used adaptive interference cancellation (AIC) algorithm, this paper develops several details to avoid these limitations and form a new mitigation architecture to alleviate nonlinear distortions. To demonstrate the efficiency of the proposed algorithm, verification tests for both simulations and actual RF front-end measurements are presented and discussed. The obtained results show that distortions can be suppressed significantly, thus improving the reliability of spectrum sensing. Moreover, compared to AIC, the proposed algorithm clearly shows better performance, especially at the band edges of the interferer signal.

## 1. Introduction

The proliferation of Micro-Electro-Mechanical Systems (MEMS) technology has facilitated the development of smart sensors, enabling wireless sensor networks (WSNs) by providing smaller, cheaper, and more intelligent sensors [1]. Current WSNs operate in the Industrial Scientific Medical (ISM) bands, which are shared by many other successful communication technologies. This overcrowds the ISM bands, limiting the development of new technologies. On the other hand, the traditional approach of fixed-spectrum assignment causes many licensed spectrum bands to be either underutilized or unutilized [2]. Cognitive radio (CR) has the ability to identify the unutilized spectrum in a licensed or unlicensed spectrum band and then utilize it opportunistically. Therefore, the CR technique is probably one of the most promising techniques for improving the efficiency of WSNs. The cognitive radio wireless sensor network (CR-WSN) can effectively mitigate the current issue of spectrum inefficiency and increase the network efficiency to some extent [3,4]. Research in this area is progressing rapidly but is still in its infancy. 

Although a number of papers, such as references [5,6,7], have been published on CR-WSN, many issues still remain to be addressed, such as the reliability of spectrum sensing. One of the main objectives of embedding a CR in a wireless sensor is to utilize the unused licensed spectrum opportunistically. Here, “opportunistically” implies that secondary users (SUs) should protect the access right of primary users (PUs) whenever necessary. However, distortion products due to strong sub-bands or carriers, stemming from the radio frequency (RF) front-end nonlinearity of sensor nodes, can easily degrade the spectrum sensing performance by causing false alarms and missed detections. A false alarm can cause spectrum under-utilization and a missed detection can cause interference with the PUs. For this reason, spectrum sensing is key to the deployment of CR-WSN. Previous works have dealt with improving the reliability of spectrum sensing by developing different sensing techniques [8,9,10,11,12,13,14]. In comparison, this work focuses on analyzing and mitigating the RF front-end nonlinearity of sensor nodes for reliable sensing.

The RF front-end of a sensor node might operate in a range where its components, such as the Low Noise Amplifier (LNA), exhibit a nonlinear behavior [15,16]. Therefore, spurious frequencies are generated in the form of harmonics, intermodulation (IM), and cross modulation (XM) [17]. As a result, the presence of strong blockers, i.e., strong signals inside or outside the sub-band of interest, produce distortion terms that affect the detection performance in other sub-bands, where weaker signals may reside [17,18]. Under such scenarios, the detection performance might be degraded, causing the CR network to either exhibit harmful interference with the PU or miss the opportunity to transmit in a vacant sub-band. Rebeiz [19] was one of the first papers to study the impact of nonlinearities on spectrum sensing performance. This was subsequently followed by [20], which first analytically derived the theoretical false alarm and detection probabilities in closed form, for both energy and cyclostationary detection in nonlinear front-ends. These probabilities were derived as functions of the sensing time, powers, and modulation types of both the blockers and the Signal of Interest (SOI).

One approach to alleviate the RF impairments is the use of digital signal processing (DSP), a procedure also known as “Dirty RF” [21]. By applying this post-correction algorithm, the RF front-end linearity can be subsequently improved in the digital domain. A system-level approach to mitigate nonlinear distortions in the digital domain, so-called adaptive interference cancellation (AIC), was first presented in [22]. Subsequently [16,23], used this approach to improve the reliability of spectrum sensing. This approach is based on the principle of feed forward mitigation, which regenerates distortions with the help of a reference model and then adaptively subtracts them from the received signal. Moreover, the applications of AIC in spectrum sensing have been presented in [19,20,23,24,25,26]. In [19], Rebeiz studied availability in the performance of spectrum sensing with/without AIC. 

Although AIC has been mainly verified by both simulations and laboratory RF measurements, some limitations still remain to be considered and form the focus of this paper. First, a bandpass/bandstop filter pair is adopted in AIC to derive the desired and reference signals, and the achievable mitigation performance depends on the filter characteristics. For this, a coarse energy detector [23] is needed to obtain spectral sensing information about the level and spectral location of the strong blocker. To provide a clean reference signal, the filters must have sufficient stopband attenuation. Thus, high filter orders and significant DSP resources may be required to implement them [27]. Moreover, filter characteristics are application-specific and depend on the actual center frequency and bandwidth of the desired and blocker signals at hand [16]. If the channel of dynamic carrier allocation varies with varying center frequencies and bandwidths, for example, for multicarrier signals, the bandsplit filters will be powerless. This is the typical scenario considered for CRs [17,23,28]. To overcome these limitations, a singular value decomposition (SVD)-based method is adopted in this paper to separate the reference signal from the rest of the spectrum. This is advisable because only strong signals create significant distortions, and the SVD-based method can extract signals according to the power level.

Second, in the reference branch, the idea of AIC is to reproduce the frequencies of distortion components by feeding the blocker into a nonlinear model, with the amplitudes and phases being further modified by an online adaptive filter (AF) stage. In a practical implementation, the effective processing of second-order, third-order and higher-order nonlinearities is carried out individually, and the corresponding AFs process each order nonlinearity parallelly. In this way, the number of independent AFs is related to the number of coefficients specified in the reference model and the filter length is related to the memory considered in the reference model [27]. For simplicity, most of the existing literature adopts the memoryless polynomial model and only considers the third-order IM [22,27]. However, in wideband applications, such a representation is not realistic. In this paper, the Volterra series [29], which is a well-known model for the analysis of nonlinear systems, is adopted. We also consider all the distortions together. To do this, we design a blind identification criterion and objective function to adjust the Volterra coefficients adaptively, so that no AF is needed. Additionally, the use of bandstop filters in AIC, for stable coefficient adaptation, prevents the cancellation of in-band nonlinear distortions on the blocker bands as well as on the bandstop filter transition bands. The phenomenon is most pronounced when using limited-order filters, which is usually the case in true implemented hardware [30]. However, in our mitigation structure, the reference branch is unfiltered.

Third, after the distortion terms are regenerated, the AF coefficients are adjusted by minimizing the power of the mitigated output using the well-known least mean square (LMS) algorithm or any of its variants [31] in nearly all the existing literature. The LMS algorithm has the problems of convergence and complexity. In addition, the achievable mitigation performance depends on the step-size of LMS. In this paper, by deriving a formula for our mitigation structure, we conclude that the Volterra coefficients can be obtained simply by solving linear equations. We emphasize that despite the fairly straightforward steps of the derivation, to our knowledge, this conclusion has not been reported earlier in the existing literature of the field. 

The outline of the remainder of this paper is as follows. Section 2 introduces the system architecture of CR-WSN and the challenges in spectrum sensing. Section 3 presents the proposed mitigation algorithm for nonlinear distortions generated by the RF front-end of sensor nodes. Then, Section 4 provides the results of simulations and RF measurements. Additionally, comparisons with AIC are provided to show the effectiveness of the proposed algorithm. Finally, conclusions are drawn in Section 5.

## 2. System Model and Problem Analysis

### 2.1. CR-WSN Architecture

**Figure 1:**
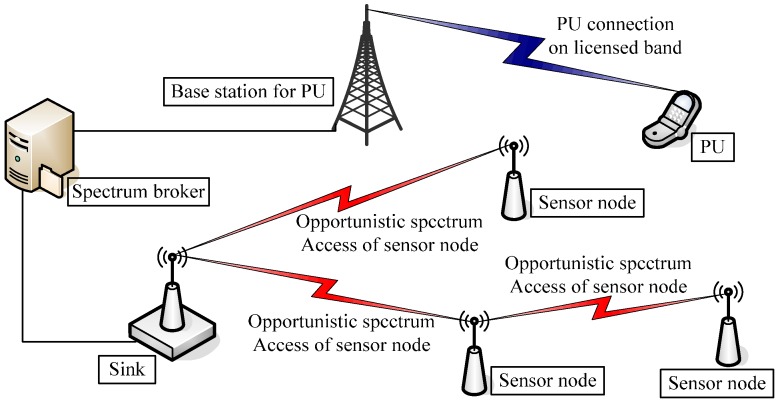
A simple cognitive radio wireless sensor network (CR-WSN) model.

Taking advantage of the current liberalization of the spectrum utilization rules by the Federal Communications Commission (FCC) and technical advancements in sensor technology, wireless sensors with CR can exhibit increased spectrum utilization and network efficiency. A typical CR-WSN consists of many base stations (BSs) and PUs, and hundreds of sensor nodes. For simplicity, a minimalistic CR-WSN model is shown in Figure 1. Depending on the spectrum availability, sensor nodes transmit their readings in an opportunistic manner to their next hop sensor nodes, and ultimately, to the sink. In addition to the event readings, the sensors may exchange additional information with the sink, including control data for group formation, spectrum allocation, and spectrum handoff-aware route determination, depending on the specific topology.

**Figure 2:**
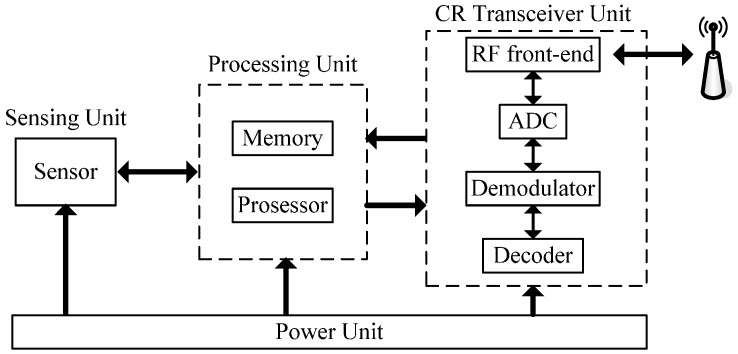
Hardware structure of a CR-WSN sensor node.

The sensor node of CR-WSN, whose main duty is to perform sensing on both the environment and the spectrum, can reconfigure its operating frequency, modulation, channel coding, and output power without hardware replacement. This is the most significant difference between CR-WSN and WSN. The hardware structure of a CR-WSN node is mainly composed of a sensing unit, processing unit, power unit, and CR transceiver unit, as shown schematically in Figure 2. The main difference between the hardware structure of classical sensor nodes [32] and CR-WSN nodes is the CR transceiver of CR-WSN nodes. For convenience, the term “sensor nodes” denotes CR-WSN nodes throughout the rest of this paper.

**Figure 3:**
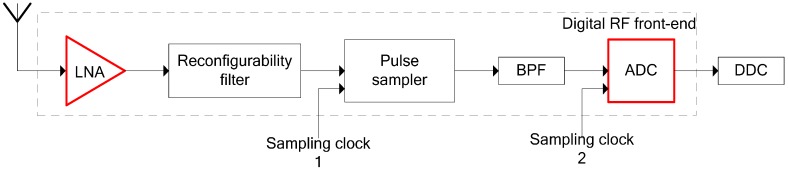
Direct-digitization radio frequency (RF) front-end block diagram highlighting the components that are considered sources of nonlinearity in this paper.

Software-defined radio (SDR)-based RF front-end transmitters and receivers are required for reconfigurability of sensor nodes. However, implementing the RF front-end is a significant challenge due to the low cost and resource-constrained nature of sensor nodes. Compared to several heterodyne or homodyne architectures, a direct-digitization RF front-end can be adopted in order to reduce costs and consumption. The structure of the direct-digitization RF front-end is illustrated in Figure 3. After the LNA stage, the desired signal band will be selected by the adjustable reconfigurability filter. By choosing an appropriate pulse sampling frequency, the signal is then down converted to a certain Nyquist band, followed by a bandpass filter (BPF), and subsequently, by an analog-to-digital converter (ADC). Finally, the baseband signal is achieved by a digital down converter (DDC), which is suitable for signal processing on the digital back-end, such as spectrum sensing and demodulation. This type of architecture can eliminate RF impairments caused by the analog mixer and the local oscillator in a conventional RF front-end, thus simplifying the hardware design with fewer analog components. Here, the main sources of nonlinear distortions are LNA and ADC, which are highlighted in Figure 3. Therefore, there is no mirror image interference and in-phase/quadrature (I/Q) imbalance. Moreover, several existing baseband post-distortion techniques [16,22] are limited to scenarios in which interferers are in the proximity of the desired channel. However, in this architecture, distortions from far interferers can also fall into the desired channel.

### 2.2. Challenges in the Spectrum Sensing Method

In the last decade, CR has been proposed as a dynamic band allocation technology, through which SUs exploit licensed spectrum holes to opportunistically access, without harmful interference to the licensed PUs. As a result, reliable spectrum sensing is key to the deployment of a future CR-WSN. At present, various sensing schemes have been proposed, e.g., energy detection (ED) [8,9,10], matched-filter-based detection [11], cyclostationary-based detection [12], and eigenvalue-based sensing [13]. These methods, which have different levels of complexity and performance, may require an estimation or a priori knowledge of noise and signal models. Such information cannot always be communicated to the receiver, especially in dynamic CR scenarios. However, ED, which detects signals on the basis of the sensed energy, is a very popular technique, not only because of its simplicity but also because it does not require prior knowledge of PUs. Therefore, in this paper, we consider ED as the detection method for analysis.

ED estimates the energy in the sub-band of interest to determine whether the SOI is present or otherwise, by comparing the estimated energy to a threshold. In this paper, we do not consider the noise uncertainty problem of ED and only show the degradation of ED due to RF front-end nonlinearities. The detector therefore computes the test statistic, given by

(1) $$R_{x}=\frac{1}{N}\sum_{k=0}^{N-1}{\left|x\left(k\right)\right|}^{2}$$

and performs the following hypothesis tests

(2) $$\{\begin{array}{cc} R_{x}<\gamma , & H_{0}:SOI\;is\;absent\\ R_{x}\geq \gamma , & H_{1}:SOI\;is\;present \end{array}$$

where *N* is the sub-band length of interest; *x*(*k*) is the sub-band signal; γ is the detection threshold that is usually set to maintain a Constant Probability of False Alarm (CFAR), i.e.,

(3) $$P_{cfar}=P_{f}\left(R_{x}\geq \gamma |H_{0}\right)$$

and γ is calculated by

(4) $$\gamma =\sigma_{w}^{2}\left(N+\sqrt{2N}Q^{-1}(P_{f})\right)$$

where $\sigma_{w}^{2}$ is the noise variance of the sub-band; *Q*^−1^(·) is the inverse function of

(5) $$Q(x)=\frac{1}{2\pi }\int_{x}^{\infty }\exp\left(-\frac{u^{2}}{2}\right)du$$

and *P_f_* is the false-alarm probability, which is a metric to quantify the failure of CR-WSN to detect the absence of the primary signal. For threshold calculations throughout the paper, a noise-only measurement and false alarm probability *P_f_* = 0.01 are used. It is noted that the threshold γ should not be constant and may change with different observations for random noise. Even in the same observation, the thresholds of different sub-bands are always different. However, for each sub-band in one observation, a concrete threshold can be calculated from (4). The thresholds shown in the figures of this paper only reflect the thresholds of the sub-bands of interest in one observation.

Although ED requires the least amount of information about the signal to be detected, it does suffer from the linearity problem due to the limited dynamic range of the RF front-end. The main objectives for considering nonlinear distortions in the operation of a CR are reliable spectrum sensing under huge dynamic range conditions, and proper demodulation of weak but desired signals for communication. The distortion impact is three-fold. First, distorted products can show up as unwanted signals in free frequency bands, where CR may operate as an SU. As a consequence, simple energy-based spectrum sensing would detect this band as occupied and the SU would miss its transmit opportunity. Second, the distortions may mask weak signals so that the detectors are not able to discover them, which increases the probability of missed detection and enables harmful transmission in busy bands. Third, nonlinear distortions can be caused on top of a weak desired signal, causing an increased bit error ratio (BER) and hence, difficulties during demodulation.

**Figure 4:**
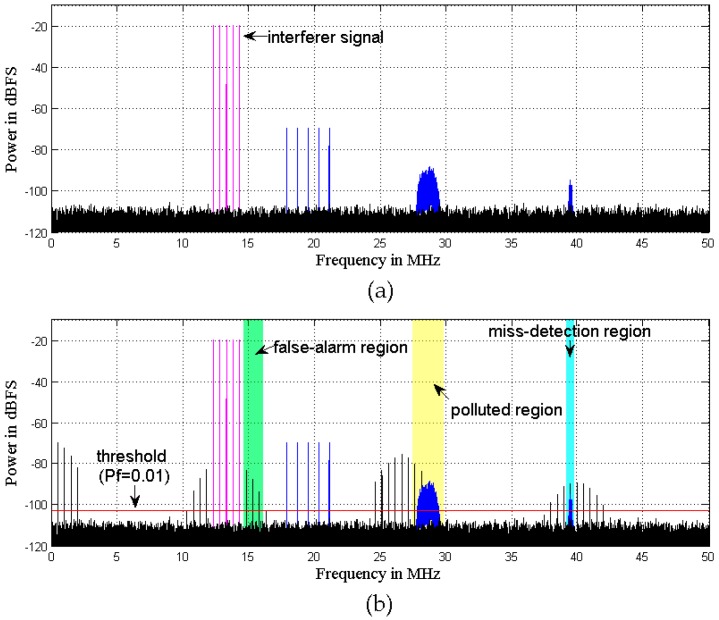
Typical simulation results of a distorted spectrum and the energy detection (ED) threshold: (**a**) original signal; (**b**) distorted signal.

Figure 4 shows an example simulation of a distorted spectrum to imitate RF front-end nonlinearities and illustrate the effects in spectrum sensing. Figure 4a gives the power spectrum of the original signal, which is comprised of an interferer signal and three weak desired signals. The power spectrum of the distorted signal is shown in Figure 4b, and the threshold in 1024 sub-bands of the so-called false-alarm region for ED is depicted. In the false-alarm region, the detected “signal present” is actually a false alarm due to IM induced by the nonlinear front-end. This results in the loss of opportunity for SU. In the so-called miss-detection region, a weak desired signal is masked by nonlinearities, resulting in missed detection. In the so-called polluted region, the distortions overlap with another weak desired signal, because of which the desired signal cannot be demodulated. All these scenarios can decrease the reliability of spectrum sensing.

In some systems, spectrum sensing problems can be avoided by using a centralized database to provide information about vacant channels [33]. However, spectrum access itself may be challenging due to the RF front-end nonlinearity. In particular, non-contiguous spectrum access is challenging because there might be strong blockers between the desired channels, causing nonlinear distortions [17]. Therefore, mitigation of RF front-end nonlinearity is expected to enhance the reliability of detection.

## 3. Proposed Mitigation Architecture for RF Front-End Nonlinearity

The digital approaches that are currently widely-used to mitigate nonlinearity are based on AIC, originally presented in [16,22]. However, most of the existing literature is characterized by the following: focus on baseband correction, adoption of a bandpass/bandstop filter pair to derive the desired and reference signals, parallel processing of nonlinear distortions of every order, adoption of the memoryless polynomial model and consideration of only third-order IM, and use of the LMS algorithm to adjust the adaptive filter coefficients. In this paper, we develop these details to avoid some of the resulting limitations, as mentioned in the introduction.

### 3.1. Mitigation Structure

**Figure 5:**
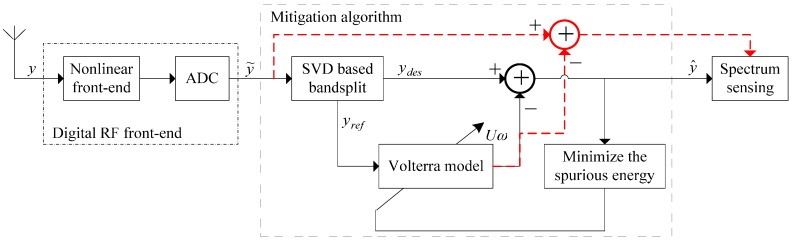
Proposed mitigation structure for RF front-end nonlinearity.

The block scheme of the proposed mitigation algorithm is illustrated in Figure 5 for the case of a spectrum sensing scenario, where the goal is to eliminate distortions from the whole reception bandwidth in order to enhance spectrum sensing [17,23]. Due to the adoption of a direct-digitization RF front-end, the mitigation algorithm works at the waveform level of a real signal rather than a complex baseband signal. Thus, the I/Q imbalance can be avoided and the algorithm structure can be simplified. The entire front-end and ADC are considered as a single nonlinear system, i.e., a black-box model. Therefore, the algorithm has no relation to the concrete structure of the front-end and the physical origins of the distortions, and can thus be inserted in front of any system-specific baseband processing.

A SVD-based method is adopted to split the distorted signal into the main branch *y_des_*, and the reference branch *y_ref_*. The latter contains only the interferer signal, i.e., the blocker, while the main branch contains all the other received signal contents except the interferer signal. The nonlinear distortion generated by the front-end is re-generated in the reference branch by applying a Volterra model with adjustable coefficients to the interferer signal. To minimize the power of the mitigated output, the Volterra coefficients can be adapted by solving linear equations. The re-generated nonlinear distortion is then subtracted from the main branch, thus compensating the nonlinear distortion in the received signal. To also receive the interferer signal, the re-generated nonlinear distortion can be directly subtracted from the received signal, as suggested by the red dashed branch in Figure 5. In the existing literature, the interferer signal is added back to the main branch, which may lead to additional distortion if the interferer signal itself is not well and truly extracted in the bandsplit stage. Finally, each sub-band of interest can be selected individually by a DDC to achieve the baseband signal for spectrum sensing.

The following analyses deal with the SVD-based method for the bandsplit stage and the adjustment of Volterra coefficients in the proposed mitigation algorithm, which are also the main contributions of this work.

### 3.2. SVD-Based Method for Bandsplit Stage

The key idea of the proposed mitigation algorithm is to extract the crucial interferers contained in the received signal and to reproduce the distortions caused by them. Therefore, the achievable mitigation performance depends on the correctness and veracity of the extracted interferer signal in the bandsplit stage. Generally, bandsplit filters need coarse energy detection and sufficient stopband attenuation. Additionally, they are powerless when faced with multi-interferers or dynamic channel allocation. It is likely that only strong signals create significant distortions. Thus, only strong interferers in the received band should be identified. The SVD-based method extracts signals according to the power level, and is therefore most suitable for separating the interferer signal.

First, the elements of the distorted signal $\tilde{y}$ are embedded into a *m* × *n* dimensional matrix

(6) $${\tilde{Y}}_{m\times n}=\left[\begin{array}{cccc} \tilde{y}\left(1\right) & \tilde{y}\left(2\right) & \cdots & \tilde{y}\left(n\right)\\ \tilde{y}\left(n+1\right) & \tilde{y}\left(n+2\right) & \cdots & \tilde{y}\left(2n\right)\\ \vdots & \vdots & \ddots & \vdots \\ \tilde{y}\left(\left(m-1\right)n+1\right) & \tilde{y}\left(\left(m-1\right)n+2\right) & \cdots & \tilde{y}\left(mn\right) \end{array}\right]$$

where the data length is N=m×n. Then, the SVD is extended to the matrix ${\tilde{Y}}_{m\times n}$

(7) $${\tilde{Y}}_{m\times n}=U_{m\times p}\Sigma_{p\times p}V_{p\times n}^{T}$$

where p=min(m,n) and the main diagonal elements $\sigma_{i}$(i=1, 2, ⋯, p) of the diagonal matrix Σ are called singular values of $\tilde{Y}$, which are non-negative and in descending order. According to the theory of SVD, larger singular values mainly reflect stronger signals. By keeping the largest *q* singular values and zeroing the rest, a new main diagonal matrix can be obtained

(8) $${\hat{\Sigma }}_{q\times q}=diag(\sigma_{1},\;\sigma_{2},\;\cdots ,\;\sigma_{q})$$

Then, the estimated interferer signal matrix can be achieved by

(9) $$Y_{m\times n}=U_{m\times q}{\hat{\Sigma }}_{q\times q}V_{q\times n}^{T}$$

Finally, the estimated interferer signal *y_ref_* can be obtained from matrix *Y*. By subtracting this from the distorted signal $\tilde{y}$, the main branch signal *y_des_* can be achieved.

According to the above SVD-based method, the performance of the extracted interferer signal is largely determined by the selection of an effective order *q*. A lower order will lead to incomplete interferer signal information, while a higher order will introduce certain nonlinearity or information about weak desired signals. Since the types of actual multicarrier signals received by the RF front-end of CR nodes are complex, it is very difficult to determine *q*. Thus far, a widely-used method relies on the asymptotic property of singular entropy increment [34]. However, there is essentially little difference between the curve shape for a singular value and singular entropy increment apart from amplitude variation. No obvious characteristics can be used to determine the effective order. 

In this paper, a method is presented according to increments of the singular value. The increment curve reflects the difference between the adjacent singular values, so that a larger increment represents a larger difference of signal strength. In general, the power of the interferer signal is far stronger than that of nonlinear distortions and the weak desired signal. Therefore, the largest difference exists at the boundary between the interferer signal and the other signals, which is shown as the minimum increment for the descending order of singular values. The increments are always zero in the noise platform. Therefore, the corresponding order of minimum increment can safely be chosen to be the effective order *q*.

**Figure 6:**
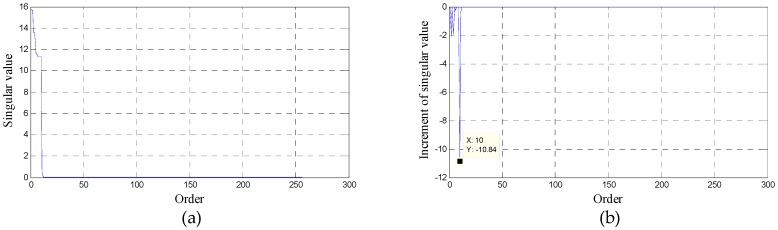

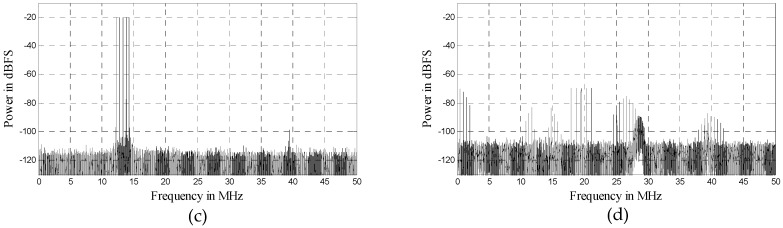
Simulation results of the signal used in Figure 4: (**a**) Curve of singular value; (**b**) Increment curve of singular value; (**c**) Power spectrum of the extracted interferer signal; (**d**) Power spectrum of the distorted signal except the interferer signal.

To evaluate the availability of the proposed methods of selecting effective order and extracting interferer signals, we take the simulation signal in Figure 4 as an example. The simulation results are shown in Figure 6. Figure 6a gives the curve of singular values and we can see that the order is hard to choose. On the other hand, in Figure 6b, it is obvious that the order can be determined easily and reliably (*q* = 10) by the increment curve. The power spectra of the extracted interferer signal and all other signals for a selected order of 10 are shown in Figure 6c,d, respectively. Clearly, good signal extraction performance is achieved.

### 3.3. Adjustment of Volterra Coefficients

The mitigation performance also depends on the degree of matching between the reference model and the real-world nonlinear behaviors of the actual RF front-end. Therefore, it is necessary to achieve accurate coefficients of the reference model. Most existing literature adopts the memoryless polynomial model and mitigates each order nonlinearity parallelly. Thus, multiple LMS AFs are needed, which may exhibit the problems of convergence and complexity. In this paper, a modified reference branch processing is explained.

The Volterra series is considered to be suitable for any nonlinear system. The input/output relationship for a nonlinear system can be expressed by the discrete Volterra model [29]

(10) $$\begin{array}{cc} v\left(k\right)=\sum_{d=2}^{D}\left[\sum_{r_{1}=0}^{N_{d}-1}\cdots \sum_{r_{d}=r_{d}-1}^{N_{d}-1}h\left(r_{1},r_{2},\cdots ,r_{d}\right)\prod_{j=1}^{d}y_{ref}\left(k-r_{j}\right)\right], & 2\leq d\leq D \end{array}$$

where $y_{ref}\left(k\right)$ is the extracted interferer signal, v(k) represents the output signal of the discrete Volterra model of the kth discrete sample, *d* is the nonlinear order, *D* is the maximum value of *d*, $N_{d}$ is the memory depth of the *d*th-order Volterra kernel, and $h\left(r_{1},r_{2},\cdots ,r_{d}\right)$ is the *d*th-order Volterra kernel coefficient, of which the total number is given by

(11) $$N_{h}=\sum_{d=2}^{D}\frac{\left(N_{d}+d-1\right)\;!}{\left(N_{d}-1\right)\;!\;d\;!}$$

Define $u\left(k\right)=[y_{ref}^{2}(k)\;y_{ref}(k)y_{ref}(k-1)\;\cdots \;y_{ref}^{2}(k-N_{d}+1)\;y_{ref}^{3}(k)\;y_{ref}^{2}(k)y_{ref}(k-1)\;\cdots \;y_{ref}^{D}(k-N_{d}+1)]\in R^{1\times N_{h}}$ as the memory nonlinear vector of each order, which consists of $y_{ref}\left(k\right)$ and $\omega ={\left[h(0,0)\;h(0,1)\;\cdots \;h(N_{2}-1,N_{2}-1)\;h(0,0,0)\;h(0,0,1)\;\cdots \;h(N_{D}-1,\cdots ,N_{D}-1)\right]}^{T}\in R^{N_{h}\times 1}$ as the Volterra kernel vector of the corresponding order and memory depth. Then, the mitigated output signal of the kth discrete sample can be written as

(12) $$\hat{y}(k)=y_{des}(k)-u(k)\omega$$

When the reference model is matched perfectly with the nonlinear distortions, the mitigated output only contains the desired signal. Therefore, a blind identification criterion for minimizing the short-time energy of the mitigated output is designed in order to construct the objective function required by the adaptive adjustment of Volterra coefficients. The short-time energy of the mitigated output is

(13) $$E(\omega )=\sum_{k=1}^{N}{\left[\hat{y}(k)\right]}^{2}={\hat{Y}}^{T}\hat{Y}={\left[Y_{des}-U\omega \right]}^{T}[Y_{des}-U\omega ]$$

where

$$\hat{Y}={\left[\begin{array}{cccc} \hat{y}\left(1\right) & \hat{y}\left(2\right) & \cdots & \hat{y}\left(N\right) \end{array}\right]}^{T}\in R^{N\times 1}$$

$$Y_{des}={\left[\begin{array}{cccc} y_{des}\left(1\right) & y_{des}\left(2\right) & \cdots & y_{des}\left(N\right) \end{array}\right]}^{T}\in R^{N\times 1}$$

$$U={\left[\begin{array}{cccc} u\left(1\right) & u\left(2\right) & \cdots & u\left(N\right) \end{array}\right]}^{T}\in R^{N\times N_{h}}$$

To minimize the objective function, take the partial derivative of E(ω) with respect to ω and let

(14) $$\frac{\partial E(\omega )}{\partial \omega }=-2U^{T}Y_{des}+2U^{T}U\omega =0$$

then, the Volterra coefficients can be obtained simply by solving the linear equation

(15) $$U^{T}U\omega =U^{T}Y_{des}$$

hence,

(16) $$\omega ={\left(U^{T}U\right)}^{-1}U^{T}Y_{des}$$

Since low-order nonlinearities are the key factors causing the deterioration of the Spurious-Free Dynamic Range (SFDR) performance of the entire digital RF front-end, the mitigation algorithm in this study only requires the Volterra model with low orders and short memory lengths. Therefore, the dimensions of *U^T^U* are small, with less resource consumption in matrix inversion. For example, when *D* = 3 and *Nd* = [4, 2], the number of Volterra coefficients is 14 and the dimensions of *U^T^U* are 14 × 14. If the condition number of *U^T^U* is very large, there are several techniques for solving such an ill-conditioned system of equations, such as the augmented system method, residue iteration algorithm, weight-iterative improvement method [35], generalized inverse algorithm, etc. In this paper, the weight-iterative improvement method is adopted for ill-conditioned situations.

The mitigated output signal can be achieved by substituting *ω* into (12). If the interferer signal is also desired, the mitigated output signal will be

(17) $$\hat{y}=\tilde{y}-U\omega$$

Based on the above analysis, we can formulate a mitigation algorithm to alleviate the nonlinear distortions of the RF front-end of sensor nodes. The concrete implementation steps are as follows:

(1) Create the distorted signal matrix $\tilde{Y}$ from distorted signal $\tilde{y}$ according to (6);
(2) Extend SVD to matrix $\tilde{Y}$;
(3) Choose the effective order q according to the minimum increment of singular values and obtain the new main diagonal matrix $\hat{\Sigma }$ from (8);
(4) Reconstruct the estimated interferer signal matrix Y according to (9), and achieve the estimated interferer signal $y_{ref}$;
(5) Achieve the main branch signal $y_{des}$ by subtracting $y_{ref}$ from $\tilde{y}$;
(6) Calculate U from $y_{ref}$ according to (10), and calculate ω from (16);
(7) Depending on the desire to retain the interferer signal or otherwise, the mitigated output signal $\hat{y}$ can be achieved by (17) or (12).

### 3.4. Complexity Analysis

CR wireless sensors are power constraint devices with a limited energy source. The cost of CR sensors, which are generally deployed in large numbers, should be very low. Therefore, energy-efficient and low-cost sensor nodes should be designed. However, the introduction of the proposed mitigation architecture inevitably causes extra energy consumption and production costs.

In addition to the energy needed for spectrum sensing, CR sensors also require energy for channel negotiation, route discovery, transmission and reception of data packets, backoff, data processing, and frequent spectrum handoff. The transmission of control information and retransmission upon failure to transmit data packets result in unnecessary energy consumption. Nonlinear distortions significantly increase the BER and interruption probability of transmission, thus increasing the above-mentioned unnecessary energy consumption. Therefore, though the nonlinearity mitigation module itself will increase the energy consumption to a certain extent, it also certainly helps to reduce the energy consumption. Furthermore, the energy consumption of CR-WSN also depends on the spectrum sensing technique, sensing strategy, spectrum decision, clustering scheme, modulation technique, etc. It is hard to obtain a qualitative and quantitative analysis of the change in energy consumption by embedding the proposed mitigation architecture. Several solutions for energy efficiency have been proposed in recent years, such as cooperative spectrum sensing [14], a distributed spectrum-aware clustering scheme, and energy harvesting.

For the above reasons, in this section, we only consider the complexity introduced by the proposed mitigation architecture, which requires extra hardware resources and cost. According to the concrete implementation steps addressed in the previous section, the computational complexity is shown as follows.

(1) Let the data length N=m×m. Extending SVD to matrix ${\tilde{Y}}_{m\times m}$ by QR decomposition (The name “QR” is derived from the use of the letter Q to denote orthogonal matrices and the letter R to denote right triangular matrices.), the required calculations include: $\frac{1}{3}\left[10m^{3}+\left(12P-5\right)m^{2}+\left(27P+23\right)m-\left(18P+60\right)\right]$ times addition, $\frac{1}{3}\left[16m^{3}+\left(24P-1\right)m^{2}+\left(36P+10\right)m-\left(30P+14\right)\right]$ times multiplication, $2m^{2}+\left(4P+4\right)m-\left(3P+10\right)$ times division, and (2P+2)m−(P+3) times square-root. Here, P is the number of QR iterations.
(2) m−1 iterations of subtraction are required to choose the effective order q.
(3) The required iterations of addition and multiplication for reconstructing the estimated interferer signal are $m^{2}\left(q-1\right)+mq\left(q-1\right)$ and $m^{2}q+mq^{2}$, respectively.
(4) To obtain U, $N\cdot \sum_{d=2}^{D}\frac{\left(N_{d}+d-1\right)\;!\left(d-1\right)}{\left(N_{d}-1\right)\;!\;d\;!}$ iterations of multiplication are needed.
(5) The required iterations of addition and multiplication of the automoment matrix $U^{T}U$ generation module are ${N_{h}}^{2}\left(N-1\right)$ and ${N_{h}}^{2}N$, respectively.
(6) The automoment matrix inversion is implemented by LU decomposition (The name “LU” is derived from the use of the letter L to denote upper triangular matrices and the letter U to denote lower triangular matrices.). The required iterations of addition and multiplication are $N_{h}\left(N_{h}-1\right)$ and ${\left(N_{h}-1\right)}^{3}$, respectively.
(7) To calculate $U^{T}Y_{des}$, $N_{h}\left(N-1\right)$ iterations of addition and $N_{h}N$ iterations of multiplication are needed.
(8) To calculate ω from (16), the required iterations of addition and multiplication are $N_{h}\left(N_{h}-1\right)$ and ${N_{h}}^{2}$, respectively.
(9) Real-time mitigation of nonlinear distortions is performed according to (17). The required iterations for both addition and multiplication are $NN_{h}$.

Table 1 shows the computational complexity statistics of the proposed mitigation architecture. For N=m×m and $N>>N_{h}$, we adopt m to be the measurement base of complexity. It is true that the proposed architecture results in significant resource consumption for large quantities of data. In engineering applications, a faster SVD algorithm can be adopted.

## 4. Simulation Experiments and Analysis of Results

This section provides the results of simulations and actual RF measurements, which show the impact of RF nonlinearity on the detection performance of ED and evaluate the performance of the proposed mitigation architecture. To the best of our knowledge, AIC is one of the most widely-used approaches for nonlinearity mitigation because of its good performance, practicability, and applicability. As described in the previous sections, the proposed architecture, which incorporates several details to avoid the limitations caused by AIC, can be considered as an improved AIC. Therefore, we also provide explicit comparisons to AIC, reported earlier in [16,23], to illustrate the superiority of the proposed algorithm. Throughout this section, we consider the interferer signal remaining in the mitigated output signal as discussed in Section 3.

### 4.1. Verification Test Using Simulation Signals 

**Figure 7:**
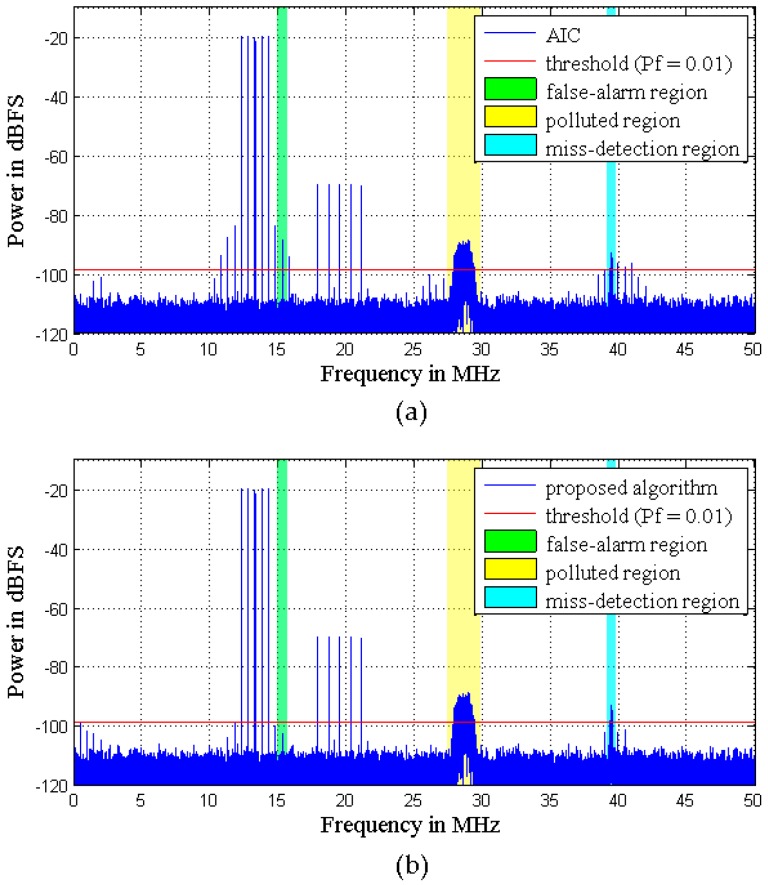
Simulation results with the signal used in Figure 4 and mitigation with (**a**) adaptive interference cancellation (AIC); (**b**) the proposed algorithm.

The first example considers a multi-tone signal constellation, as sketched in Figure 4, which is comprised of an interferer signal and a weak signal. The interferer is a five-tone sine at 13.3 MHz with −20 dB (500 kHz tone spacing). The weak signal is a combined signal of a five-tone sine at 19.5 MHz with −69.5 dB (800 kHz tone spacing), a 16-QAM (quadrature amplitude modulation) at 28.6 MHz with −89 dB (1 MHz bandwidth), and another weak 16-QAM at 39.4 MHz with −95 dB (200 kHz bandwidth). The sample rate is 100 MHz. The signal and carrier frequencies are chosen arbitrarily, the only condition being that the 16-QAM signals should be located in distortion regions. In the proposed algorithm, the power spectra of the extracted signals are shown in Figure 6, and a Volterra model with *D* = 3 and *N_d_* = [2, 2] is adopted. To demonstrate the advantages of the SVD-based bandsplit method and Volterra coefficients adjustment method, the same model is adopted in AIC. Moreover, in AIC, the order of the filter pair is 100 and the number of iterations of LMS is 600. Figure 7 illustrates how well the mitigation algorithms are able to remove the distortions. While both mitigation algorithms perform adequately, the algorithm proposed here shows slightly better overall performance.

**Figure 8:**
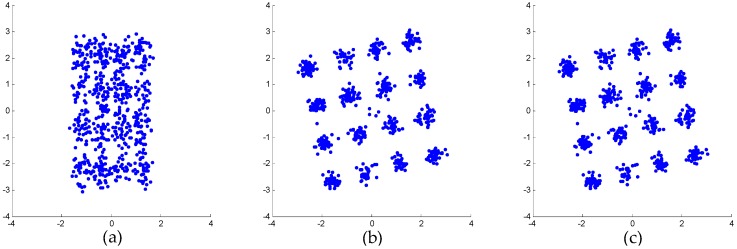
Demodulated constellations of the 16-QAM (quadrature amplitude modulation) signals in the polluted regions: (**a**) unmitigated signal; (**b**) output of AIC; (**c**) output of the proposed algorithm.

Specifically, the weak 16-QAM signals in the miss-detection regions are clearly observable from the output spectra of both mitigation algorithms, i.e., the detection probability of ED is improved. In the polluted regions, the 16-QAM signals can be demodulated well by both mitigation algorithms, as shown by the constellations in Figure 8. This means that the reception performance of weak signals is enhanced. However, in the false-alarm regions, a spectrum hole can be found after the proposed algorithm while a false alarm is still observed after AIC. This is because AIC uses a bandpass filter to the pick interferer signal and the mitigation is not perfect in the filter transition bands. In fact, Figure 7b clearly shows that the mitigated output of the proposed algorithm is almost clear of unwanted RF components in the whole band. In comparison, in Figure 7a, the mitigated output of AIC still presents a few distortion components above the ED threshold for *P_f_* = 0.01.

Figure 8 shows that the 16-QAM signals are indeed better after mitigation. For the whole multi-tone simulation signal, the normalized mean squared error (NMSE) can be considered as a metric to evaluate the efficiency of the proposed algorithm. NMSE is defined as

(18) $$NMSE=10\mathrm{lg}\left[\frac{\sum_{n=1}^{N}{\left|\hat{z}(n)-z(n)\right|}^{2}}{\sum_{n=1}^{N}{\left|z(n)\right|}^{2}}\right]$$

where z(n) is the known ideal signal; for explicit comparisons of the mitigation performance, $\hat{z}(n)$ is set to be the distorted signal, the output of AIC and the output of the proposed algorithm, respectively. Because the mitigated power of nonlinear distortions is extremely small relative to the power of the interferer signal, the interferer signals are not contained in both z(n) and $\hat{z}(n)$ for the calculations of NMSE presented here.

**Figure 9:**
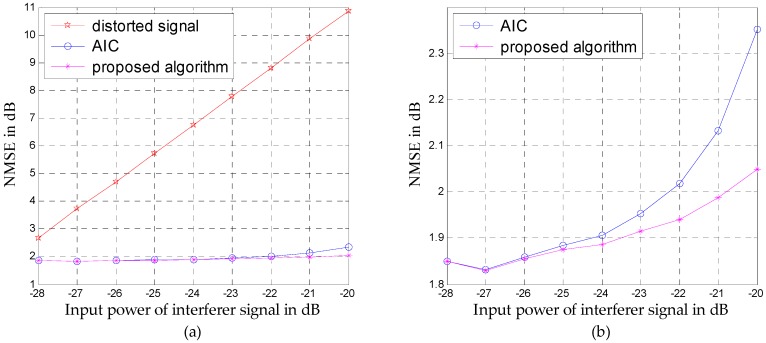
Normalized mean squared errors (NMSEs) of the weak desired signals at different input power levels: (**a**) comparison of the unmitigated signal with the outputs of AIC and the proposed algorithm; (**b**) comparison between the outputs of AIC and the proposed algorithm.

Since the generated nonlinear distortions increase with increasing input power of the interferer signal, the obtained NMSEs at different input power levels for the above simulation signal are illustrated in Figure 9. Figure 9a shows that both mitigation algorithms can improve the desired weak signals greatly. The magnified view in Figure 9b shows that the proposed algorithm performs slightly better than AIC, especially in the presence of stronger distortions.

**Figure 10:**
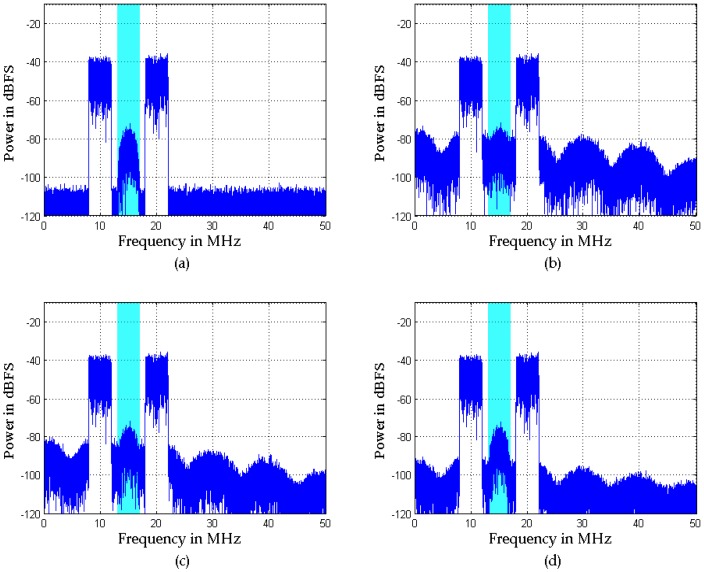
Simulation results with the two-tone interferer signal: (**a**) original signal; (**b**) distorted algorithm; (**c**) output of AIC; (**d**) output of the proposed algorithm.

To further demonstrate the impact of nonlinear distortions on the miss-detection of ED, we give another example in which the weak signal is almost masked by the distortions of the interferer signal, as shown in Figure 10b. In Figure 10a, the interferer signal is a two-tone 4-MHz-wide blocker signal at center frequencies of 10 MHz and 20 MHz, and the weak signal is a 16-QAM at 15 MHz with −73 dB (2 MHz bandwidth). The sample rate is 100 MHz. It is evident from the magenta spectra of the distorted signal that the reception of weak signals is impossible due to the vast quantity of nonlinear distortions. Similarly, a Volterra model with *D* = 3 and *N_d_* = [2, 2] is adopted in both mitigation algorithms. In AIC, the bandsplit stage filters have to be designed with two passbands. The order of the filter pair is 500 and the number of iterations of LMS is 1000. 

**Figure 11:**
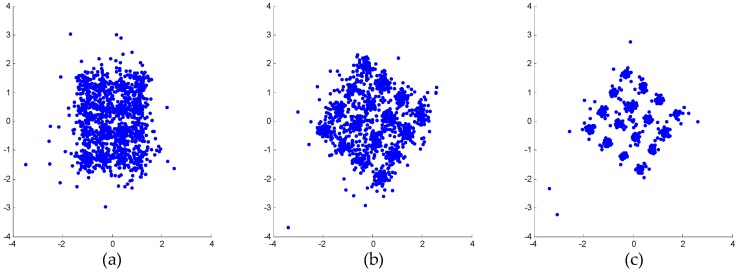
Demodulated constellations of the weak 16-QAM signals: (**a**) unmitigated signal; (**b**) output of AIC; (**c**) output of the proposed algorithm.

Figure 10c shows the mitigated result of AIC. It can be seen that the weak 16-QAM signal can be detected, but the performance is poor, as shown by the constellations in Figure 11b. Figure 10d and Figure 11c illustrate that the performance of the proposed algorithm is better than that of AIC. This is also because the reference signal extraction in AIC suffers from in-band distortions of the interferer signal, which causes degraded mitigation performance at interferer band edges due to the bandpass filter transition bands. 

**Figure 12:**
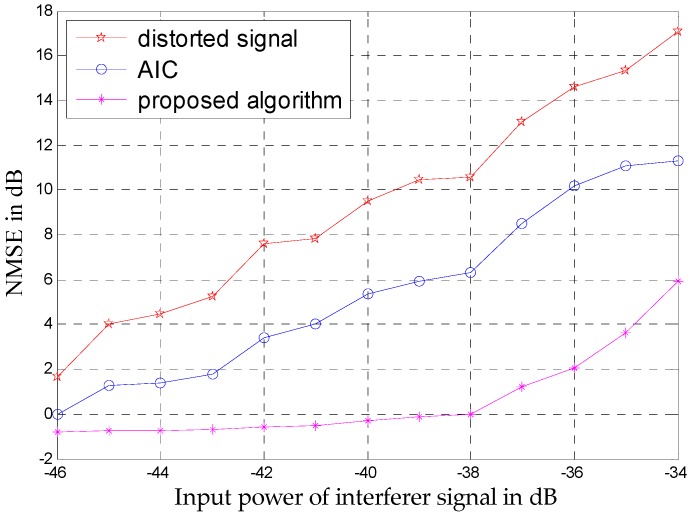
NMSEs of the weak 16-QAM at different input power levels for the unmitigated signal and the outputs of both AIC and the proposed algorithm.

To evaluate the efficiency of the proposed algorithm, NMSE is also used in this example. Similarly, the NMSEs of the weak 16-QAM are obtained by adjusting the input power levels of the two-tone interferer signal. As shown in Figure 12, both mitigation algorithms can improve the 16-QAM to a great degree, but the improvement of the proposed algorithm is much larger than that of AIC.

### 4.2. Verification Test Using Actual RF Measurements

Real-world measurements were also conducted to demonstrate and verify the mitigation capabilities of the proposed algorithm with true RF signals and components. A vector signal generator with satisfactory SFDR was used to generate clean test signals, which were delivered to a direct-digitization RF front-end to produce nonlinear distortions.

**Figure 13:**
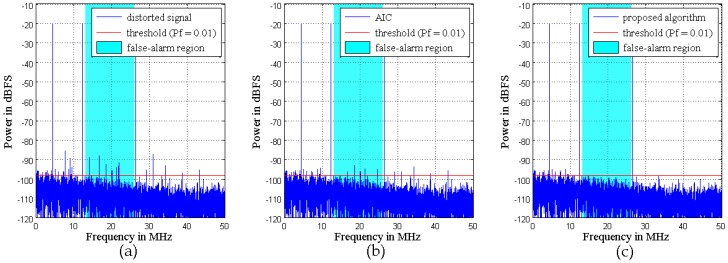
Simulation results with a three-tone interferer signal: (**a**) distorted signal; (**b**) mitigated output of AIC; (**c**) mitigated output of the proposed algorithm.

Figure 13 illustrates the mitigation performance achieved with a three-tone input. The tone frequencies are 4.5 MHz, 12.3 MHz, and 26.4 MHz, and the power is −20 dB. The sample rate is 100 MHz. A Volterra model with *D* = 3 and *N_d_* = [6, 4] is adopted in both mitigation algorithms. In AIC, the bandsplit stage filters have to be designed with three extremely narrow passbands with sufficient stopband attenuation. Therefore, the order of the filter pair is set to 2000 and the number of iterations of LMS is 4000. Unlike the simulation signals, there is no knowledge about the noise variance. For threshold calculation, a noise-only signal is captured beforehand. The used front-end indicates good linearity with mild nonlinear distortions. However, both mitigation algorithms can still alleviate distortions dramatically. With the proposed algorithm in particular, there are almost no more unwanted components present above the noise floor in the whole band.

**Figure 14:**
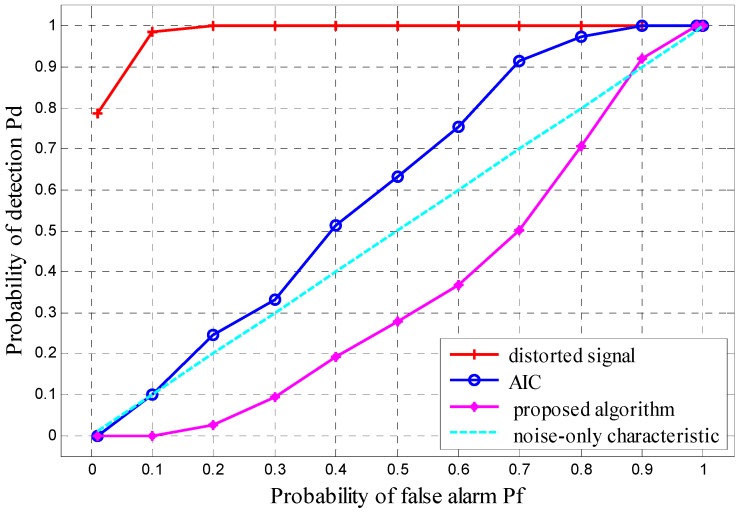
Receiver operating characteristics (ROC) before and after mitigation by different algorithms.

To further demonstrate the performance improvement of the mitigation algorithms for spectrum sensing, the cyan band in Figure 13 is chosen as the band of interest for analysis. Figure 14 illustrates the receiver operating characteristics (ROC) for each case, showing the detection of spurious signals. For the distorted signal, the spurious signal detection probability *P_d_*, or equivalently, the false alarm probability due to distortions is always one, except for *P_f_* ˂ 0.2. This means that ED will always detect this band as occupied and SU will miss its transmit opportunity. The ROC curve of the AIC output is highly improved, but is still always above the ideal noise-only characteristic (*P_d_* ≈ *P_f_*). In other words, certain distortions still exist in this band, especially at large *P_f_*. In comparison, the ROC curve of the mitigated output of the proposed algorithm is always below the ideal noise-only characteristic, indicating that there are no more distortions in this band. The effect of nonlinear distortions is fully compensated, thus improving the reliability of spectrum sensing.

**Figure 15:**
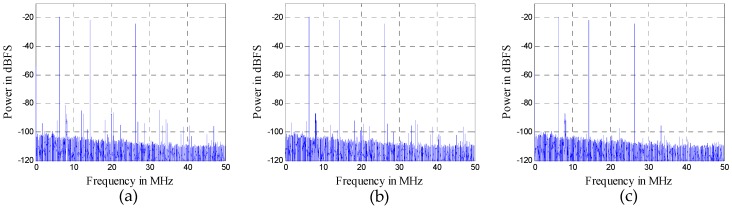
Simulation results with a 3-tone interferer signal with a weak 16-QAM signal: (**a**) distorted signal; (**b**) output of AIC algorithm; (**c**) output of the proposed algorithm.

We give another example to demonstrate the impact of nonlinear distortions on the reception capability of weak signals. A signal composed of a three-tone signal (the frequencies are 6.3 MHz, 14.3 MHz, and 26.3 MHz) and a weak 16-QAM signal (the center frequency is 8.1 MHz, and the bandwidth is 0.25 MHz) is used, as shown in Figure 15. The power spectrum value of the 16-QAM signal is approximately 65 dB lower than that of the three-tone signal, and the sample rate is 100 MHz. A Volterra model with *D* = 3 and *N_d_* = [8, 4] is adopted in both mitigation algorithms. In AIC, the order of the filter pair is 2000 and the number of iterations of LMS is 6000. Obviously, AIC is more complex.

**Figure 16:**
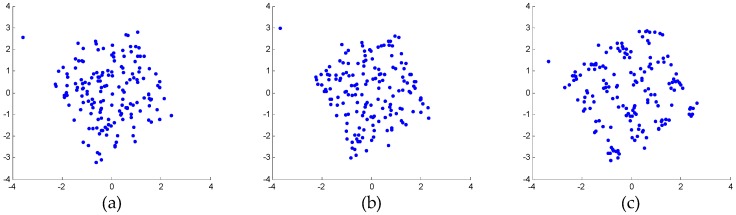
Demodulated constellations of the weak 16-QAM signals: (**a**) unmitigated signal; (**b**) output of AIC algorithm; (**c**) output of the proposed algorithm.

In view of both the power spectra shown in Figure 15 and the constellations shown in Figure 16, the proposed algorithm provides better performance than AIC. As shown in Figure 16, the distorted signal and the AIC output signal cannot be demodulated. On the other hand, the output signal of the proposed algorithm can be demodulated. Although the convergence effect in Figure 16c is also not very good, this is because the signal-to-noise ratio (SNR) of the weak 16-QAM signal itself is not high enough. In other words, the reception performance of weak signals is enhanced by the proposed mitigation algorithm.

In summary, mitigation algorithms can improve the reliability of spectrum sensing in CR-WSN from three aspects: reduce the false alarm probability, enhance the reception performance of weak signals, and decrease the miss-detection probability. From the verification tests of both simulations and actual RF front-end measurements, the proposed algorithm clearly outperforms AIC, especially at the interference signal band edges. Therefore, it is justifiable to say that the proposed mitigation algorithm is suitable and effective in CR applications for enabling flexible sensing and processing of the RF spectrum.

## 5. Conclusions 

Rather than developing different sensing techniques, in this paper, we consider ways to mitigate the RF front-end nonlinearity of sensor nodes to improve the reliability of spectrum sensing in CR-WSN. Compared to the widely-used AIC algorithm, the main advantages of the proposed mitigation algorithm are as follows:

(1) A SVD-based bandsplit method is adopted instead of using bandpass/bandstop filter pairs. Thus, the coarse energy detector is not needed to achieve spectral sensing information about the level and spectral location of strong blocking signals. More importantly, the SVD-based method can effectively avoid the problem of poor compensation performance at blocker band edges due to the bandpass filter transition bands.
(2) The proposed algorithm adopts the Volterra model and obtains the Volterra coefficients simply by solving a linear equation. There is no need to use AFs, and the selection of the iteration step-size as well as the problems of the convergence and complexity of LMS can be circumvented.

In summary, the proposed algorithm can improve the reliability of spectrum sensing and outperforms the AIC algorithm. It is predicted to have broad and promising applications in CR-WSN. However, a major challenge in a CR-WSN is to produce low-cost, very small sensor nodes. In other words, a single sensor node cannot sustain very complicated signal processing. This further development offers an interesting and relevant topic for our future studies.

## Figures and Tables

**Table 1:** Computational complexity of the proposed mitigation architecture.

Operator	Computational Complexity
Addition	$O\left(m^{3}\right)$
Multiplication	$O\left(m^{3}\right)$
Division	$O\left(m^{2}\right)$
square-root	O(m)

## Abbreviations

The following abbreviations are used in this manuscript:

CR-WSN	Cognitive Radio Wireless Sensor Network
RF	Radio Frequency
AIC	Adaptive Interference Cancellation
SVD	Singular Value Decomposition
MEMS	Micro-Electro-Mechanical Systems
WSN	Wireless Sensor Network
ISM	Industrial Scientific Medical
CR	Cognitive Radio
SU	Secondary User
PU	Primary User
LNA	Low Noise Amplifier
IM	Intermodulation
XM	Crossmodulation
SOI	Signal of Interest
DSP	Digital Signal Processing
AF	Adaptive Filter
LMS	Least Mean Square
FCC	Federal Communications Commission
BS	Base Station
SDR	Software-Defined Radio
BPF	Bandpass Filter
ADC	Analog-to-Digital Converter
DDC	Digital Down Converter
I/Q	In-phase/Quadrature
ED	Energy Detection
CFAR	Constant Probability of False Alarm
BER	Bit Error Ratio
SFDR	Spurious-Free Dynamic Range
QAM	Quadrature Amplitude Modulation
NMSE	Normalized Mean Squared Error
ROC	Receiver Operating Characteristics
SNR	Signal-to-Noise Ratio
